# Consistent condom use increases spontaneous regression in high-risk non-HPV16 but not in HPV16 CIN2-3 lesions, a prospective population-based cohort study

**DOI:** 10.1186/1750-9378-7-30

**Published:** 2012-11-05

**Authors:** Ane Cecilie Munk, Irene Tveiterås Øvestad, Einar Gudlaugsson, Kjell Løvslett, Bent Fiane, Bianca van Diermen-Hidle, Arnold-Jan Kruse, Ivar Skaland, Emiel AM Janssen, Jan PA Baak

**Affiliations:** 1 Department of Obstetrics and Gynecology, Stavanger University Hospital, Stavanger, Norway; 2 Department of Pathology, Stavanger University Hospital, Box 8100, 4068, Stavanger, Norway; 3 Department of Gynecology, Academic Hospital Maastricht, Maastricht, The Netherlands

**Keywords:** CIN2-3, High-risk HPV, HPV16, Regression, Condom use, Clinical factors

## Abstract

**Background:**

The major cause of cervical intraepithelial neoplasia (CIN) is persistent infection with human papillomavirus (HPV). Most CIN grade 2 and 3 lesions are treated with cone excision, although a substantial proportion (6-50%) of CIN2-3 lesions will regresses spontaneously. Predictors for regression of CIN2-3 are desirable in order to reduce this overtreatment.

**Methods:**

In this prospective cohort study, 145 consecutive women with first-time onset CIN2-3 in colposcopy-directed biopsies and standardized biopsy-cone excision interval were included. The genotype of the high-risk human papillomaviruses (=*hr*HPV) and clinical factors including sexual behaviour, parity, contraception and smoking were assessed. Patients were divided into two groups according to lesions containing HPV16 (*hr*HPV16+) and high-risk non-HPV16 (*hr*HPV16-) genotypes.

**Results:**

Women whose partners consistently used condoms showed a significantly higher regression rate than women using other types of contraception (53% versus 13%, p<0.0001). However, this effect was only seen in *hr*HPV16- patients (73% regression rate versus 13%, p<0.0001). *Hr*HPV16+ patients had a significantly higher number of sexual partners and more current smokers compared to *hr*HPV16- patients. The regression rate was not significantly different in CIN2-3 lesions containing HPV16 (*hr*HPV16+) versus *hr*HPV16- genotypes.

**Conclusions:**

Heterogeneity among *hr*HPV genotypes excists. HPV-genotype analyses can identify women who significantly increase their chance of regression by consistent condom use.

## Background

Cervical intraepithelial neoplasia (=CIN) is the premalignant condition of invasive cervical cancer caused by persistent infection with HPV, which can be detected in over 99% of high-grade CINs and cervical cancers [1,2]. HPV is the most common sexually transmitted agent worldwide [3,4] and up to 80% of all women will be infected with genital HPV during life [3,5]. Most infections clear within 1–2 years [6,7], but a minority of women with high-risk HPV will develop CIN [8,9]. CIN lesions are dynamic, which means they can progress to invasive cancer, persist for many years or regress spontaneously [10,11], depending on the balance between the virus and host factors such as the individual local immune response [12,13]. Several risk factors for development of CIN have been identified, including sexual behaviour, parity, contraception type and smoking [6,14-17]. However, there are only a few studies on regression in relation to already established CIN and HPV genotypes [13,18-20].

The standard treatment of punch-biopsy detected high-grade CIN2-3 is cone excision, although only about 30% eventually progress to cancer [21]. A substantial proportion (6-50%) of CIN 2–3 regress spontaneously over time depending on diagnostic criteria and follow-up time [10,11,18]. Cone excision is an invasive procedure, carrying the risk of potential complications [22]. The most serious complication is cervical insufficiency in a future pregnancy, leading to a higher risk of late abortion and preterm delivery during the second and early third trimester of pregnancy [23,24]. Furthermore, current screening programs and conventional therapeutic guidelines lead to considerable and increasing over-treatment of CIN [25]. Due to the lack of known factors, which could predict or promote regression of CIN 2–3, many women are treated unnecessarily with cone excision, although many would have regressed spontaneously within months [18,26].

Different *hr*HPV genotypes have different carcinogenic potential. HPV16 is the most common *hr*HPV genotype found in more than 50% of cervical cancers [27]. Further, HPV16 is the most carcinogenic one with the highest risk of CIN2-3 lesions progressing to invasive cancer and a lower probability of regression compared to other genotypes [13,28,29].

The aim of this prospective study was to examine potential associations between *hr*HPV genotypes, regression rate and clinical factors such as sexual behaviour, parity, condom use and smoking in women with first-time onset CIN2-3 and standardized long interval (median 16 weeks) between punch biopsy and cone excision [18].

## Methods

The study has been approved by the Norwegian Regional Ethical committee (#NR303.06), the Social and Health Department (#07/3300) and the Norwegian Social Science Data Service (#17185). All patients gave written informed consent.

### Gynecologic and pathology methods

Two hundred and fifty-four women aged 25–40 years, who were referred to the gynecology outpatient clinic at Stavanger University Hospital for evaluation of atypical cytological cervical smear between January 2007 and December 2008, were consecutively included. Norwegian guidelines for examination and reporting of atypical cytology were followed: 1. Low grade Squamous Intraepithelial Lesion (LSIL) or 2. Atypical Squamous Cells of Undetermined Significance (ASCUS) + *hr*HPV positivity, 3. Recurrences of *hr*HPV positive cervical smears, 4. Atypical Squamous Cells, can not rule out a High-grade lesion (ASC-H) and 5. High Grade Squamous Intraepithelial Lesion (HSIL).

**Figure 1:**
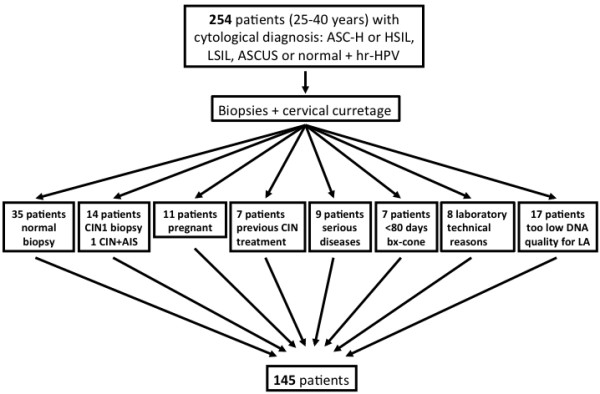
**Inclusion and exclusion criteria.** Norwegian guidelines for histological examination of atypical cytology were followed: 1. Low grade Squamous Intraepithelial Lesion (LSIL) + high-risk Human Papillomavirus (hr-HPV) or 2. Atypical Squamous Cells of Undetermined Significance (ASCUS) + hr-HPV or 3. Recurrences of hr-HPV positive cervical smears or 4. Atypical Squamous Cells, cannot rule out a High Grade lesion (ASC-H) or 5. High Grade Squamous Intrepithelial Lesion (HSIL). AIS denotes Adenocarcinoma in situ and Bx Biopsy

One hundred and nine patients were excluded due to various reasons (Figure 1). Follow-up consisted of at least 3 visits to the gynecology outpatient clinic.

At the 1^st^ visit patient characteristics and clinical data regarding age, number of sexual partners, age at first sexual intercourse, sexual activity span (= interval between first sexual intercourse and age at study inclusion), parity, contraception and smoking were registered. A colposcopy was performed prior to punch biopsies and endo-cervical curettage.

At the 2^nd^ visit (week 7–9) a second colposcopy was performed to detect any fast developing premalignant mucosal changes in the transformation zone.

At the 3^rd^ visit (between 12 and 24 weeks) patients' use of contraceptives since the baseline visit was assessed and interval between biopsy and cone excision calculated. Consistent condom use was defined as those women, whose partners used condoms for all instances of sexual intercourse in the interval between biopsy and cone excision. Finally, after a third colposcopy a cone excision of the abnormal area was performed using a loop electrosurgical excision procedure. Cone excision after more than 16 weeks after punch biopsy was related to patient delay.

According to standard operating procedures punch biopsies and conisation material were fixed in 4% buffered formaldehyde (24–48 h) at 20 °C and embedded in 56 °C paraffin. All fixed biopsies were carefully oriented by using Eosin-mediated embedding, which allows macroscopic identification of the epithelium and reduces erroneous orientation of the biopsy in the paraffin block and thereby tangential cutting.

The Norwegian national guidelines for histological grading of cervical dysplasia recommend to follow the CIN classification which classify low-grade dysplasia or cervical intraepithelial neoplasia grade 1 (CIN1) as equivalent to cytological LSIL. Moderate-dysplasia or CIN2 and severe dysplasia/carcinoma in situ or CIN3 as equivalent to cytological HSIL [30]. The diagnosis of each CIN grade is based on well-defined histopathological criteria [31]. Standard Hematoxylin Erythrosin Saffran (HES) sections were used for histological evaluation and independently reviewed by two experienced gynecological pathologists (EG, JB), who were blinded for each other’s diagnosis. The proliferation marker Ki-67 and the tumour suppressor protein p16 were used to optimize the diagnoses [13,32]. Regression was defined as a CIN2–3 diagnosis in the cervical biopsy and CIN1 or less in the subsequent cone.

### HPV- analysis

DNA- material from all biopsies at inclusion was isolated (E.Z.N.A.^TM^ Tissue DNA Kit, Omega Bio-Tek, Inc., Norcross, GA, USA). HPV analyses were performed using Linear Array (LA) HPV Genotyping test (Roche Molecular Systems, Roche Diagnostics, Mannheim, Germany), which detects 37 different HPV genotypes. The LA primers amplify HPV-DNA from 16 high-risk (16, 18, 31, 33, 35, 39, 45, 51, 52, 56, 58, 59, 68, 73, 82 and IS39, which is a subtype of 82), 3 possible high-risk (26, 53 and 66), 9 unclassified (55, 62, 64, 67, 69, 71, 83, 84 and 89) and 9 low-risk genotypes (6, 11, 40, 42, 54, 61, 70, 72 and 81) in addition to β-globin DNA as a cellular control. The amplification step was performed according to the Roche users’ manual as previously described [13]. Two observers, using the Linear Array HPV Genotyping Test Reference Guide, manually interpreted the LA HPV genotyping strips [13]. All HPV genotyping was done after the diagnostic part of the study and had no influence on the follow up of the patients.

Patients were divided into two groups according to the presence or absence of HPV16. The *hr*HPV16+ group was positive for HPV16 genotype independent of other genotypes, while the *hr*HPV16- group was negative for HPV16 but positive for other high-risk genotypes.

### Statistical analysis

SPSS, version 19 (SPSS Inc., Chicago, IL, USA) was used for statistical analyses. Data are presented as median with range unless otherwise stated. The Kolmogorov-Smirnov test and visual inspection of plots were used to test for normal distribution. Continuous data were analysed by 2-sided t-test or Mann–Whitney U-test, as appropriate.

Receiver operating curve (ROC) analysis (MedCalc Software, Mariakerke, Belgium) was used to calculate cut-off values of the clinical factors: age at inclusion, interval between biopsy and cone excision, age at first sexual intercourse, number of lifetime partners and sexual activity span according to regression versus non-regression [33]. Chi-Square tests were performed to compare categorical variables.

A binary logistic multivariate regression model was applied to perform multivariate analyses. Probabilities < 0.05 were considered as statistically significant.

## Results

The study population consisted of 145 women with a first-time onset atypical cytological smear and histologically proven CIN2-3. All patients were positive for high-risk or possible high-risk HPV in the punch biopsy (Table 1). No patient was lost to follow-up.

**Table 1:** The different high-risk HPV genotypes are listed in the left column. The right column contains the number of patients with only one or multiple genotypes, respectively

**HPV genotype**	**Number of patients with one/multiple genotypes**
16	32 / 22
18	6 / 4
31	12 / 8
33	9 / 4
35	4 / 4
39	0 / 5
45	3 / 1
51	2 / 1
52	7 / 0
58	4 / 0
26,53,56,59,66,68,73,82	11 / 6

Genotyping revealed that 54 patients were *hr*HPV16+ and 91 patients *hr*HPV16-. The overall regression rate in the study was 18%. The regression rate in the *hr*HPV16- group (20%) was higher compared to the *hr*HPV16+ group (15%), but the difference was not statistically significant. In an additional analysis comparing regression versus non-regression in single and multiple *hr*HPV-genotypes infected lesions, the regression rates were 22% and 11%, respectively, a none-significant difference (p=0.10).

When comparing the two groups in a univariate analysis, *hr*HPV16+ patients had a significantly higher number of sexual partners and more current smokers compared to *hr*HPV16- patients (Table 2). In a multivariate analysis both numbers of sexual partners and smoking remained statistically different between groups (p=0.006 and p=0.03, respectively). Age, interval between biopsy and cone excision, distribution of CIN2 and CIN3, parity, age at first sexual intercourse, sexual activity span and condom use were not significantly different between the two *hr*HPV groups.

**Table 2:** **Clinical variables in *hr*HPV16 + versus *hr*HPV16- patients**

**Variables**	**Number n=145 (%)**	**HPV16+ n=54 (%)**	**HPV16- n=91 (%)**	**p-value**
Regression	26 (18)	8 (15)	18 (20)	0.5
Non-regression	119 (82)	46 (85)	73 (80)	
**Age**				
(median, min.-max., years)	31 (25-41)	32 (25-41)	30 (25-41)	1.0
≤30	66 (45)	20 (37)	46 (51)	0.1
>30	79 (55)	34 (63)	45 (49)	
**Interval biopsy-cone**				
(median, min.-max., days)	113 (84-171)	107(84-154)	113 (84-171)	0.6
≤115	101 (70)	38 (70)	63 (69)	0.9
>115	44 (30)	16 (30)	28 (31)	
**CIN diagnosis**				
CIN2	26 (18)	7 (13)	19 (21)	0.2
CIN3	119 (82)	47 (87)	72 (79)	
**HPV genotype**				
Single	91 (63)	32 (60)	59 (65)	0.5
Multiple	54 (37)	22 (40)	32 (35)	
**Smoking**				
Yes	48 (33)	25 (46)	23 (25)	0.009
No	97 (67)	29 (54)	68 (75)	
**Number of sexual partners**				
1-9	67 (46)	16 (30)	51 (56)	0.002
>10	78 (54)	38 (70)	40 (44)	
**Parity**				
Yes	92 (63)	36 (67)	56 (62)	0.5
No	53 (37)	18 (33)	35 (38)	
**Age of first sexual intercourse**				
≤15	38 (26)	15 (28)	23 (25)	0.7
>15	107 (74)	39 (72)	68 (75)	
**Sexual activity span**				
≤13	64 (44)	21 (39)	43 (47)	0.3
>13	81 (56)	33 (61)	48 (53)	
**Condom use**				
Yes	17 (12)	6 (11)	11 (12)	0.9
No	128 (88)	48 (89)	80 (88)	

^1^min.-max.: minimum, maximum.

Among all women whose partners used condoms for all instances they had intercourse between biopsy and cone excision (= consistent condom use), the regression rate was significantly higher compared to women using other types of contraception or whose partners inconsistently used condoms (9/17, 53% versus 17/128, 13%). However, although consistent condom use was associated with a significantly higher regression rate in the *hr*HVP16- group (73% versus 13%), this effect was not seen in *hr*HPV16+ patients (regression rate 16% versus 15%). Clinicopathologic features between the 17 consistent condom users and other patients did not differ significantly.

In *hr*HPV16- patients, age ≤15 years at first sexual intercourse, was associated with a significantly lower regression rate (4%) compared to patients with a sexual debut >15 years (25%) (Table 3). However, in a multivariate analysis only consistent condom use remained as a significant predictor for regression with an odds ratio of 19 (95% C.I. 4–82, p< 0.0001). Other factors such as age, interval between biopsy and cone excision, single or multiple *hr*HPV infections, number of sexual partners, sexual activity span, parity and smoking did not significantly affect regression rate in the *hr*HVP16- group (Table 3). In the *hr*HPV16+ group none of the examined factors was significantly correlated to regression (Table 4).

**Table 3:** **Clinical variables in *hr*HPV16- patients and regression versus non-regression**

**Variable**	**Number n=91 (%)**	**Regression n=18 (%)**	**Non-regression n=73 (%)**	**p-value**
**Age**				
(median, min.-max., years)	30 (25-41)	29 (25-41)	30 (25-41)	0.8
≤30	46 (50)	10 (22)	36 (78)	0.6
>30	45 (50)	8 (18)	37 (82)	
**Interval biopsy-cone**				
(median, min.-max., days)	113 (84-171)	108 (84-127)	113 (84-171)	0.1
≤115	63 (69)	14 (22)	49 (78)	0.4
>115	28 (31)	4 (14)	24 (86)	
**CIN diagnosis**				
CIN2	19 (21)	5 (26)	14 (74)	0.4
CIN3	72 (79)	13 (18)	59 (82)	
**HPV genotype**				
Single	59 (65)	13 (22)	46 (78)	0.5
Multiple	32 (35)	5 (16)	27 (84)	
**Smoking**				
Yes	23 (25)	2 (9)	21 (91)	0.1
No	68 (75)	16 (24)	52 (76)	
**Number of sexual partners**				
1-9	51 (56)	11 (22)	40 (78)	0.6
>10	40 (44)	7 (18)	33 (82)	
**Parity**				
Yes	56 (62)	10 (18)	46 (82)	0.6
No	35 (39)	8 (23)	27 (77)	
**Age of first sexual intercourse**				
≤15	23 (25)	1 (4)	22 (96)	0.03
>15	68 (75)	17 (25)	51 (75)	
**Sexual activity span**				
≤13	43 (47)	11 (26)	32 (74)	0.2
>13	48 (53)	7 (15)	41 (85)	
**Condom use**				
Yes	11 (12)	8 (73)	3 (27)	<0.0001
No	80 (88)	10 (13)	70 (87)	

^1^min.-max.: minimum, maximum.

**Table 4:** **Clinical variables in *hr*HPV16+ patients and regression versus non-regression**

**Variable**	**Number n=54 (%)**	**Regression n=8 (%)**	**Non-regression n=46 (%)**	**p-value**
**Age**				
(median, min-max, years)	31 (25-41)	31 (25-41)	31 (25-41)	0.9
≤30	20 (37)	1 (5)	19 (95)	0.1
>30	34 (63)	7 (20)	27 (80)	
**Interval biopsy-cone**				
(median, min-max, days)	113 (84-171)	107 (84-154)	113 (84-171)	0.1
≤115	38 (70)	6 (16)	32 (84)	0.7
>115	16 (30)	2 (12)	14 (88)	
**CIN diagnosis**				
CIN2	7 (13)	2 (29)	5 (71)	0.3
CIN3	47 (87)	6 (13)	41 (87)	
**HPV genotype**				
Single	32 (59)	7 (22)	25 (78)	0.08
Multiple	22 (41)	1 (5)	21 (95)	
**Smoking**				
Yes	25 (46)	5 (20)	20 (80)	0.3
No	29 (54)	3 (10)	26 (90)	
**Number of sexual partners**				
1-9	16 (30)	1 (6)	15 (94)	0.3
>10	38 (70)	7 (18)	31 (82)	
Parity				
Yes	36 (67)	6 (17)	30 (83)	0.6
No	18 (33)	2 (11)	16 (89)	
**Age of first sexual intercourse**				
≤15	15 (28)	2 (13)	13 (87)	0.8
>15	39 (72)	6 (15)	33 (85)	
**Sexual activity span**				
≤13	21 (39)	1 (5)	20 (95)	0.1
>13	33 (61)	7 (21)	26 (79)	
**Condom use**				
Yes	6 (11)	1 (16)	5 (83)	0.9
No	48 (89)	7 (15)	41 (85)	

^1^min.-max.: minimum, maximum.

## Discussion

This prospective study examined the influence of *hr*HPV genotypes on the regression rate. Further, potential interactions of *hr*HPV genotypes and clinical factors like age, interval between biopsy and cone excision, single or multiple *hr*HPV infections, number of sexual partners, age at first sexual intercourse, sexual activity span, parity, consistent condom use and smoking were evaluated.

The main finding was that consistent condom use significantly increased the regression rate in HPV16-, but not in *hr*HPV16+ lesions. Further, the number of sexual partners was higher and current smoking was more prevalent in *hr*HPV16+ than in *hr*HPV16- patients, with both differences reaching statistical significance both in univariate (Table 2) and multivariate analyses.

In the study population as a whole only consistent condom use was significantly associated with higher regression rates. However, the regression rate overall was not significantly different between *hr*HPV16+ and *hr*HPV16- patients.

HPV16 has been described as the genotype with the highest carcinogenic potential, the highest risk for progression to CIN3 and cervical cancer and the highest attribution to cervical cancer worldwide [29,34-36]. HPV16 infections also tend to last longer than infections caused by other *hr*HPV types [6]. However, the current study did not find a significantly lower regression rate in *hr*HPV16+ versus *hr*HPV16- patients, which is in line with previously published data [18,20,37]. There was a relatively small (5%) difference in regression rate between the two *hr*HPV-genotype groups investigated in the current study. Based on a retrospective sample size calculation a study of 906 patients per group would be needed to detect this difference with a power of 80%.

A higher number of sexual partners in *hr*HPV16+ women has also been stated in other studies [19,38]. This could be explained by the fact that increased sexual contact with new partners increases the risk of being infected with HPV16 compared to other *hr*HPV genotypes, as HPV16 is the most frequent genotype [35]. A significant association between lower age at sexual debut and lower regression rate was found in *hr*HPV16- patients only.

Smoking is counted as a risk factor for CIN development [6,17,39], but data on the effect of smoking in relation to HPV genotype are sparse. Previous studies have shown that cotinine and nicotine metabolites, which potentially have mutagenous effects on the cervical epithelium, accumulate in the cervical mucus of active cigarette smokers [40,41]. As current smoking was more prevalent among *hr*HPV16+ patients, smoking could potentiate the mutagenic effects of *hr*HPV16 resulting in an increased risk of developing CIN. Another potential explanation for the higher prevalence of smokers among *hr*HPV16+ patients is the association of smoking as a lifestyle combined with a higher-risk sexual behavior [42]. Both more sexual partners and smokers among *hr*HPV16+ patients could indicate that these risk factors are related to lifestyle.

Data indicate that condom use can have a positive effect on CIN regression [33,37]. Condom use reduces the repeated exposure of the cervical mucosa to HPV and the directly immunosuppressive effect of semen on the cervical epithelium. These factors strengthen the local immune system against the HPV infection and may promote CIN regression [43,44]. Another interesting hypothesis is that the latex of the condoms stimulates a general immune response, which might be beneficial in the clearance of HPV.

In *hr*HPV16- patients consistent condom use increased the regression rate significantly compared to none condom users. Additionally, age >15 years at first sexual intercourse was associated with a significantly higher likelihood of regression. None of the examined clinical factors had any significant effect on the regression rate in *hr*HPV16+ patients. This underlines the heterogeneity among *hr*HPV genotypes and that *hr*HPV16+ CIN lesions may behave differently compared to other *hr*HPV genotypes. *Hr*HPV16+ lesions seem to be less susceptible to cofactors related to sexual behavior and consistent condom use.

Heterogeneity of high-grade CIN related to HPV16 has previously been described in a study by Wentzensen et al., which showed that *hr*HPV16+ lesions were associated with lower mean age, worse colposcopic appearance and a higher number of lifetime sexual partners compared to *hr*HPV16- lesions [19].

As consistent condom use does not seem to affect regression rate of *hr*HPV16+ CIN lesions in contrast to *hr*HPV16- lesions, the behaviour and character of HPV16 and the subsequent immune reaction could be different compared to non-HPV16 high-risk genotypes. Recent studies have shown interesting results comparing epithelial and immune biomarkers in HPV16+ lesions versus HPV16- CIN2-3 lesions in relation to regression or not [13,20,45].

A better understanding of the different behaviour of HPV genotypes could contribute to a more individualized follow-up and treatment of CIN2-3 patients than today’s standard treatment with cone excision of all CIN2-3 lesions.

Consistent use of condoms by the male partners of the women as contraception was rather infrequent. However, the current results showing that consistent condom use by sexual intercourse increases the chances for regression significantly with an odds ratio of 19 in *hr*HPV16- lesions, could motivate a substantial proportion of women and their partners to use condoms for a limited time period.

The strength of the current study is the prospective design and the histological definition of CIN regression. The study population was relatively homogenous due to the inclusion criterion of age between 25 and 40 and the standardized interval between biopsy and cone excision. Additionally, both CIN2/CIN3 and consistent condom use was equally distributed across the two HPV groups. All 145 patients had *hr*HPV positive DNA in the punch biopsies by LA. This strengthens the diagnosis of the current biopsies, and ensures the genotype of the actual HPV infection. Stavanger University Hospital is the only hospital in the region, making this a population-based cohort study with low selection bias.

The observational period of the study, however, was relatively short in relation to the natural history of CIN. On the other hand, a longer observational period of high-grade CIN regression with the risk of progression could not have been justified at the start of the study. With current data new trials with a longer interval between punch biopsy and cone excision are acceptable in women under 40 years of age with a first time onset CIN2-3 lesion. Further, the sample size is rather moderate, which limits the separate interpretation of clinical factors in each group.

## Conclusions

In conclusion, consistent condom use significantly increased the regression rate in *hr*HPV16- but not in *hr*HPV16+ lesions. This suggests different immunologic response, and might have clinical impact, as HPV genotyping can identify patients who would significantly benefit from consistent condom use. Further heterogeneity is expressed by a higher number of sexual partners and more current smokers in *hr*HPV16+ patients.

## Abbreviations

ASC-H: Atypical Squamous Cells: cannot rule out a High-grade lesion; ASC-US: Atypical Squamous Cells of Undetermined Significance; CIN: Cervical Intraepithelial Neoplasia; HES: Hematoxylin Eosin Safran; HPV: Human Papillomavirus; hr-HPV: high-risk Human Papillomavirus; HSIL: High Grade Squamous Intraepithelial Lesion; LSIL: Low grade Squamous Intraepithelial Lesion; ROC: Receiver Operating Curve.

## Competing interests

The authors declare that they have no competing interests.

## Authors’ contributions

ACM: Involved in the conception and design of the study, performed inclusion, follow-up and collection of clinical data on the study patients, involved in the data analyses, performed writing of the manuscript. ITØ: Performed the HPV genotyping, involved in the data analyses and interpretation, provided critical revisions of the manuscript. EG: Involved in the conception and design of the study, responsible for all the histological diagnoses. KL: Involved in the conception and design of the study, contributed to clinical supervision concerning the study patients. BF: Involved in the conception and design of the study, contributed to clinical supervision concerning the study patients, provided critical revisions of the manuscript. BDH: Involved in the conception and design of the study, involved in database set-up, performed HPV genotyping. AJK: Involved in the conception and design of the study, provided critical revisions of the manuscript. IS: Involved in the conception and design of the study, responsible for all the immunohistochemical stainings, provided critical revisions of the manuscript. EAJ: Involved in the conception and design of the study, involved in the data analyses, substitution in writing the manuscript and critical revisions of the manuscript. JPB: Principal investigator, involved in the conception and design of the study, reviewed the histological diagnoses, involved in the data analyses, substitution in writing the manuscript and final approval of manuscript. All authors read and approved the final manuscript.
